# Supplementary material for: A neuromorphic bionic eye with filter-free color vision using hemispherical perovskite nanowire array retina
Source: Nat Commun. 2023 Apr 8;14:1972. doi: 10.1038/s41467-023-37581-y (PMC10082761; doi:10.1038/s41467-023-37581-y)
Supplement: Supplementary file 1 — Supplementary Information [file 41467_2023_37581_MOESM1_ESM.pdf]

**Supplementary Information for**

# A neuromorphic bionic eye with filter-free colour vision using hemispherical perovskite nanowire array retina and adaptive optics

*Zhenghao Long[1,2,3], Xiao Qiu[1,2,3], Chak Lam Jonathan Chan[1], Zhibo Sun[1,2], Zhengnan Yuan[1,2], Swapnadeep Poddar[1,2,3], Yuting Zhang[1], Yucheng Ding[1,2,3], Leilei Gu[4], Yu Zhou[1,2,3], Wenying Tang[1], Abhishek Kumar Srivastava[1,2], Cunjiang Yu[5], Xuming Zou[6], Guozhen Shen[7], Zhiyong Fan[1,2,3,8]*

[1]Department of Electronic and Computer Engineering, The Hong Kong University of Science and Technology; Clear Water Bay, Kowloon, Hong Kong SAR, China.

[2]State Key Laboratory of Advanced Displays and Optoelectronics Technologies, HKUST, Clear Water Bay, Kowloon, Hong Kong SAR, China

[3]Guangdong-Hong Kong-Macao Joint Laboratory for Intelligent Micro-Nano Optoelectronic Technology, HKUST, Clear Water Bay, Kowloon, Hong Kong SAR, China

[4]Qingyuan Research Institute, School of Electronic Information and Electrical Engineering, Shanghai Jiao Tong University, No. 800 Dongchuan Road, 200240, Shanghai, China

[5]Department of Engineering Science and Mechanics, Department of Biomedical Engineering,

Department of Materials Science and Engineering, Materials Research Institute, Pennsylvania State University, University Park, PA 16802, USA

[6]Key Laboratory for Micro/Nano Optoelectronic Devices of Ministry of Education & Hunan Provincial Key Laboratory of Low-Dimensional Structural Physics and Devices, School of Physics and Electronics, Hunan University, Changsha 410082, China

[7]School of Integrated Circuits and Electronics, Beijing Institute of Technology, Beijing 100081, China.

[8]Department of Chemical and Biological Engineering, The Hong Kong University of Science and Technology; Clear Water Bay, Kowloon, Hong Kong SAR, China.

✉Corresponding author: eezfan@ust.hk

This file include:

Supplementary Fig. 1-24

Supplementary Table 1

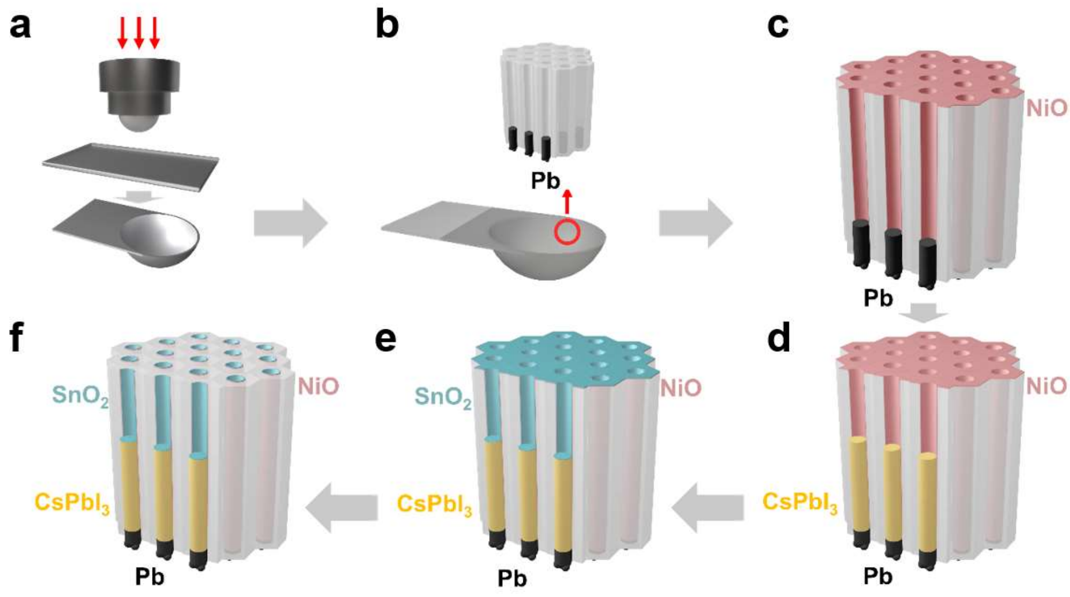


**Supplementary Fig. 1. Fabrication process of self-powered neuromorphic retina.**
**a,** Deforming of hemispherical aluminum substrate. **b,** Anodization of PAM, barrier thinning and electrochemical deposition of Pb nano clusters. **c,** ALD deposition of NiO layer. **d,** CVD growth of $CsPbI^3$ nanowire arrays and **e,** ALD deposition of $SnO_2$ layer. And **f,** ion milling process to remove NiO and $SnO_2$ on the top surface.

Firstly, A hemispherical PAM was fabricated based on a hemispherical aluminum foil via a twostep hard anodization process. Then Pb nanoclusters are synthesized at the bottom of the PAM through barrier thinning and electrochemical deposition method. Next, the PAM with Pb clusters was lifted off from the Al film in a wet etching process. The 5 nm NiO layer was deposited via atomic layer deposition (ALD). And the $CsPbI_3$ nanowires are prepared by chemical vapor deposition (CVD). Afterwards, a 5 nm $SnO_2$ layer was deposited on the device via ALD, followed by a 50 nm Au evaporation process to deposit the top electrode. After preparing the PAM, Ni micro needles based bottom electrodes were integrated on the device and connected to following measurement equipment through PCBs. Finally, the device is integrated with ionic liquid and adaptive optics.

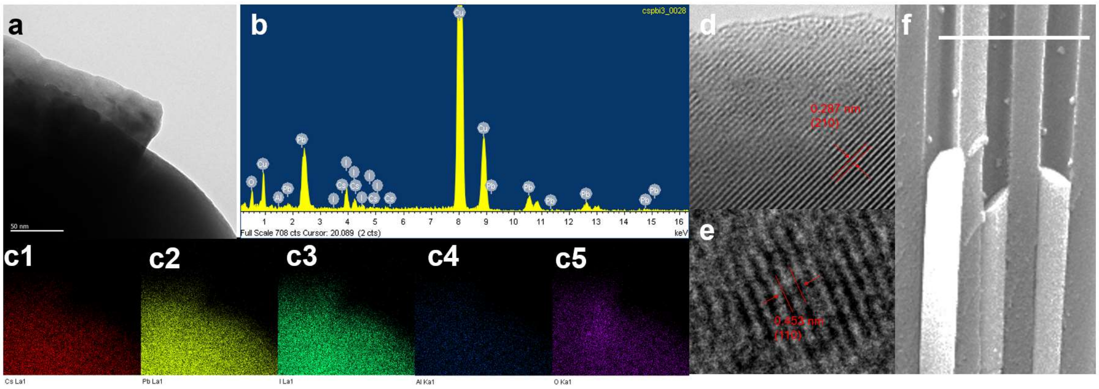


**Supplementary Fig.2. Material characterization of $CsPbI_3$ nanowires**

**a,** TEM image, **b,** Energy dispersive X-ray (EDX) analysis of $CsPbI_3$ nanowires. Elemental mapping of **c1**, Cs, **c2,** Pb, **c3,** I, **c4**, Al and **c5,** O. TEM image $CsPbI_3$ nanowire along **d,** (210) and **e,** (110) directions. And **f,** scanning electron microscope image of $CsPbI_3$ nanowires with NiO and $SnO_2$ nanotubes, the scare bar refers to 1 μm.

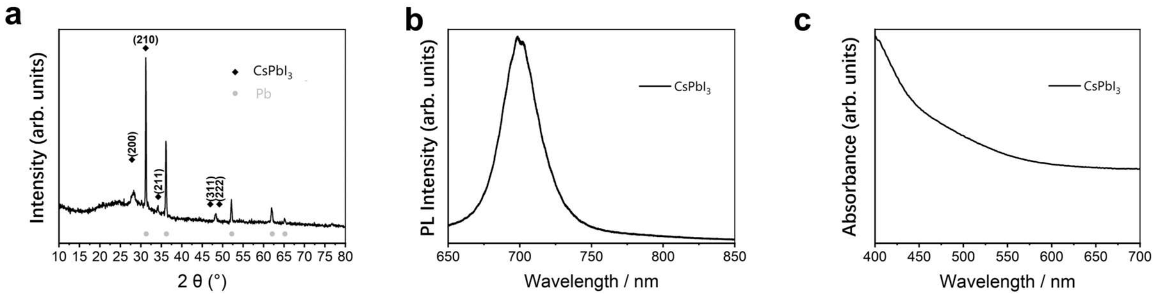


**Supplementary Fig. 3. Crystal structure and opto-electronic characterization of $CsPbI_3$ NW**

**a,** X-ray diffraction (XRD) of the $CsPbI_3$ nanowire array with Pb nanoclusters. **b,** Photoluminescence (PL) curve of the $CsPbI_3$ nanowire array for a lasing emission wavelength of 405 nm. **c,** Wavelength dependent absorbance of $CsPbI_3$ nanowire

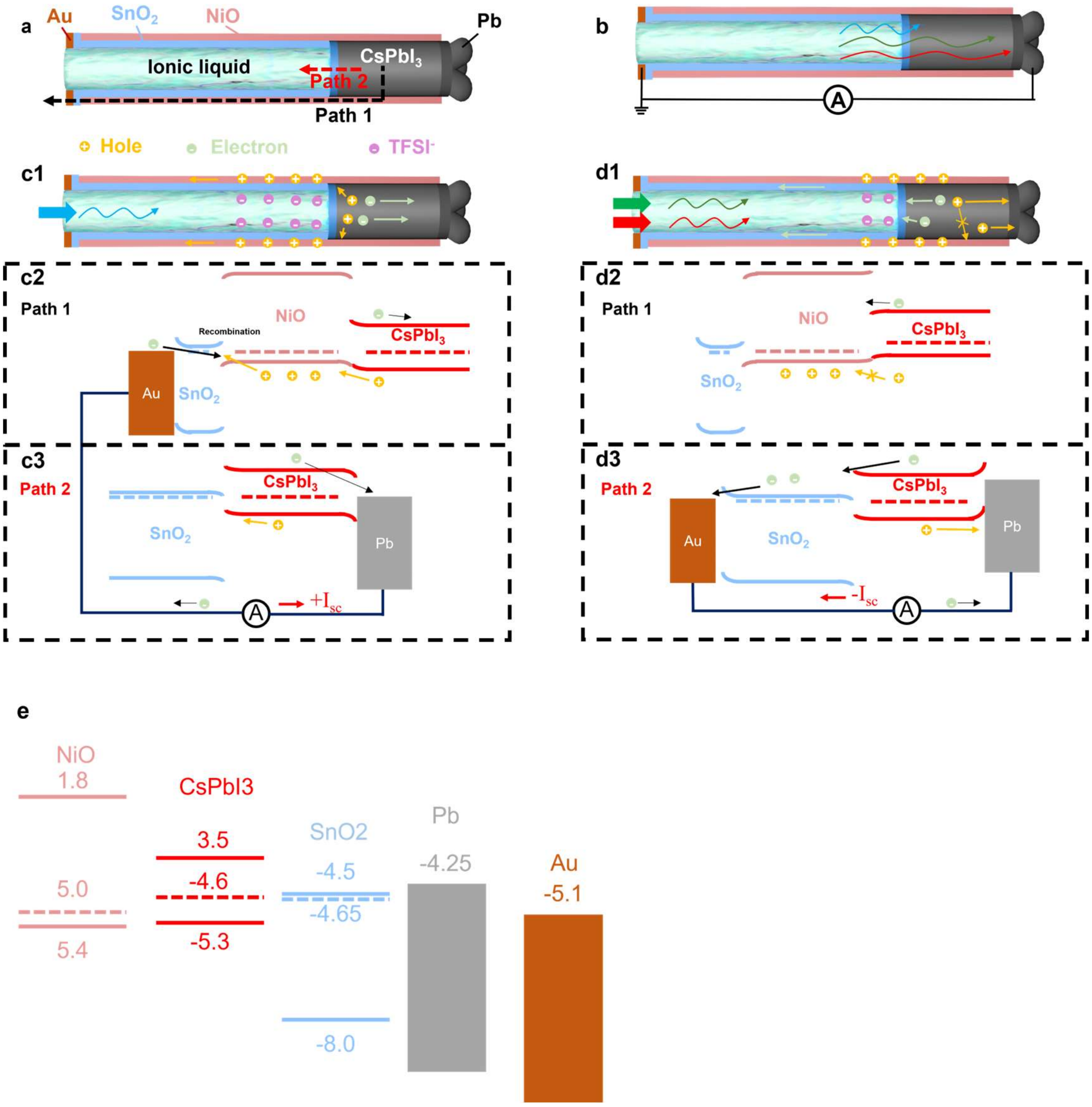


**Supplementary Fig. 4. Schematics of working mechanism and energy diagrams.**

**a,** Device structure schematic showing two carrier transportation paths. b, Schematic of circuit connection and colour illumination absorption. **c1-c3,** working mechanism under 405 nm illumination. **c1,** overall carrier motion. **c2**, energy band diagram in path 1. **c3**, energy band diagram in path 2. **d1-d3,** working mechanism under 520 nm and 650 nm illuminations. **d1,** overall carrier motion. **d2**, energy band diagram in path 1. **d3**, energy band diagram in path 2. **e,** information of energy bands, Fermi levels and metal work functions.

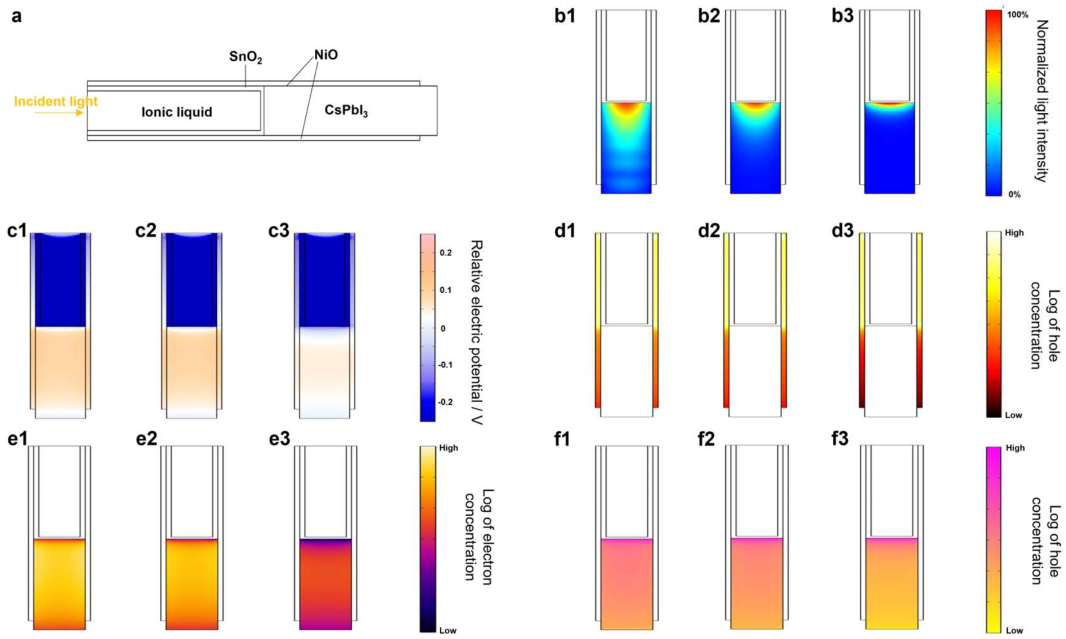


**Supplementary Fig. 5. COMSOL simulation of carrier concentration under certain illumination in perovskite nanowire**

**a,** Schematic of nanowire structure. **b1-b3,** light intensity distribution in $CsPbI_3$ of the device under **b1,** 650 nm, **b2,** 520 nm, and **b3,** 405 nm illumination, respectively. **c1-c3**, relative electric potential of the device under **c1,** 650 nm, **c2,** 520 nm, and **c3,** 405 nm illumination, respectively. **d1-d3,** hole concentration in NiO of the device under **d1,** 650 nm, **d2,** 520 nm, and **d3,** 405 nm illumination, respectively. **e1-e3,** electron concentration in $CsPbI_3$ of the device under **e1,** 650 nm, **e2,** 520 nm, and **e3,** 405 nm illumination, respectively. **f1-f3,** hole concentration in $CsPbI_3$ of the device under **f1,** 650 nm, **f2,** 520 nm, and **f3,** 405 nm illumination, respectively.

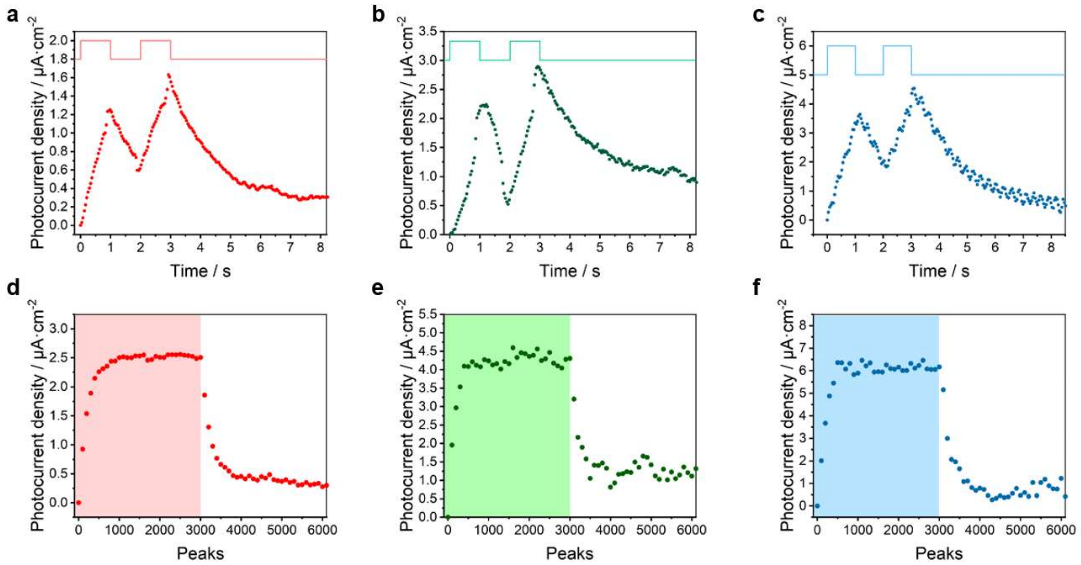


**Supplementary Fig. 6. Self-powered synaptic photo current measurement of 2 μm $CsPbI_3$ nanowire based device**

Photo current under **a,** 4.88 mW/cm$^2$ 650 nm, **b,** 4.93 mW/cm$^2$ 520 nm, and **c,** 6.59 mW/cm$^2$ 405 nm illumination with pulse width of 1 s. Photo current under 100 Hz **d,** 4.88 mW/cm$^2$ 650 nm, **e,** 4.93 mW/cm$^2$ 520 nm, and **f,** 6.59 mW/cm$^2$ 405 nm illumination. Device was exposed to illumination during 0-3000 peaks and covered by shatter during 3000-6000 peaks.

With 2 μm $CsPbI_3$ nanowire, carriers generated on the top of the nanowire under red, green and blue illumination. Thus, the device produce positive directional synaptic photo current.

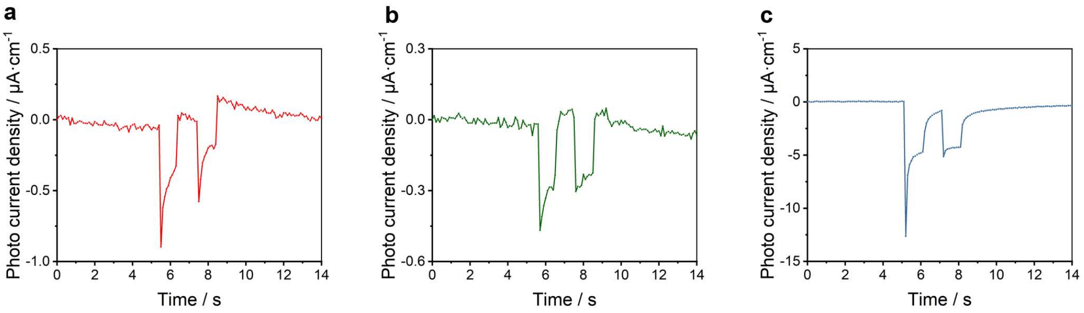


**Supplementary Fig. 7. Self-powered synaptic photo current measurement of 1 μm $CsPbI_3$ nanowire based device without ionic liquid**

Photo current under **a,** 4.88 mW/cm$^2$ 650 nm, **b,** 4.93 mW/cm$^2$ 520 nm, and **c,** 6.59 mW/cm$^2$ 405 nm illumination with pulse width of 1 s.

Without ionic liquid to balance the positive charges in NiO, the potential of $CsPbI_3$ will increase under illumination. Photo-electrons and holes move to $SnO_2$ and Pb, respectively, to generate a negative directional photo current. As there is a Schottky barrier between the $SnO_2$ and Au top electrodes, electrons can be accumulated in $SnO_2$. Without ionic liquid to balance the negative charges in $SnO_2$-Au interface, following electron transporting will be suppressed. Thus, the negative photo current gradually decreases under illumination. Specially, as blue light can generate carriers on the top of the nanowire, more electrons can be transported to the $SnO_2$ and generate stronger negative directional photo current.

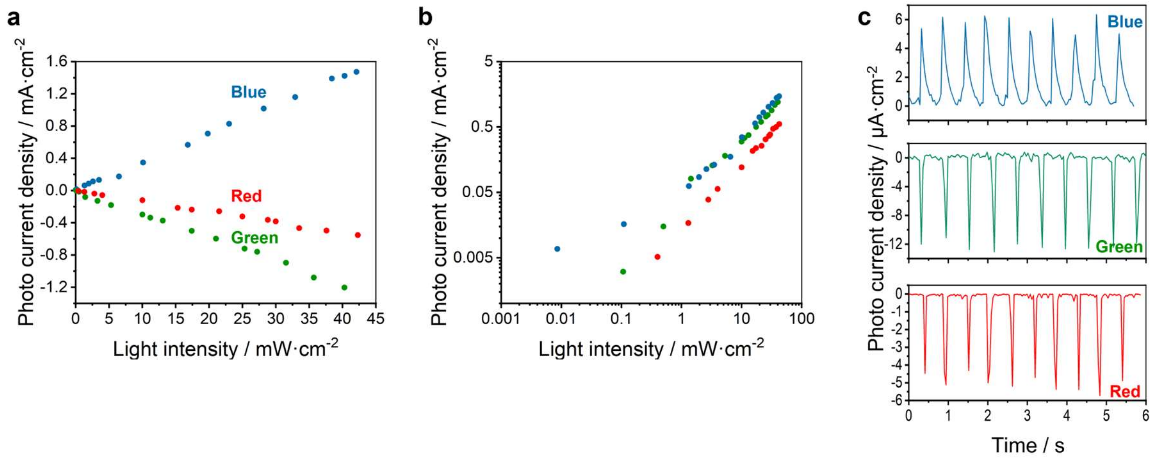


**Supplementary Fig. 8.**

**a-b,** power density dependent self-powered photo response in **a,** linear and **b,** log scale. **c,** self-powered photo response under ~2 Hz, 11mW/cm$^2$ optical stimuli.

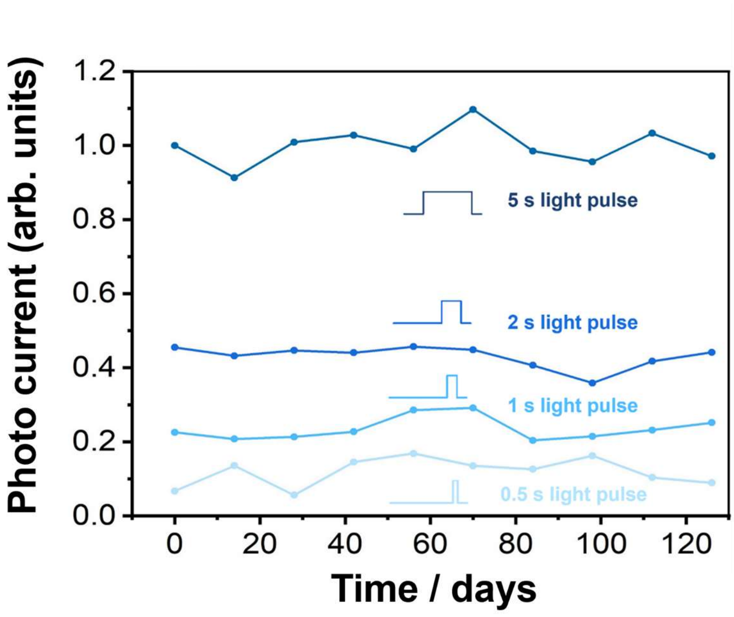


**Supplementary Fig. 9. Stability measurement of perovskite nanowire based synaptic multi-colour retina.**

Chromatic optical stimuli generated by a projector, the light intensity ~100 mW/cm$^2$. The device was kept in an indoor environment where temperature varied from 0-30 °C, and humidity very from 30-90%.

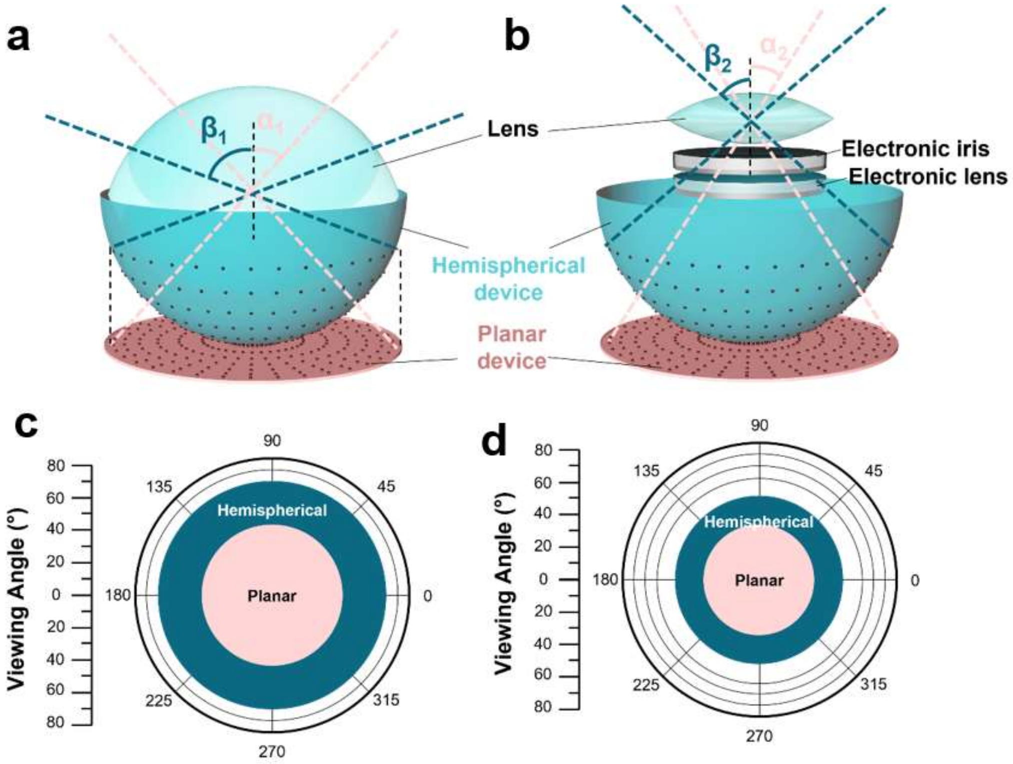


**Supplementary Fig. 10. Viewing angle and FOV calculation**

Imaging system **a,** only contains a fixed lens and an imaging device (hemispherical or plannar), along with imaging system **b,** integrated with adaptive optics. Viewing angle calculation of the hemispherical retina and planar device. The planar device is the projection of hemispherical device. **a-b** The viewing angle calculation schematics of the image sensors with a fixed lens **a,** 0 cm and **b,** 1.4 cm (The nearest position considering the thickness of electronic lens and iris) away from the center of the hemispherical device and planar device, respectively. **c-d,** The related viewing angle calculation result **c,** and **d,** respectively. Note that the FOV of hemispherical and planar devices with fixed lens shown in a are 140° and 86°, respectively. And the FOV of hemispherical and planar devices shown in b are 103° and 68°, respectively.

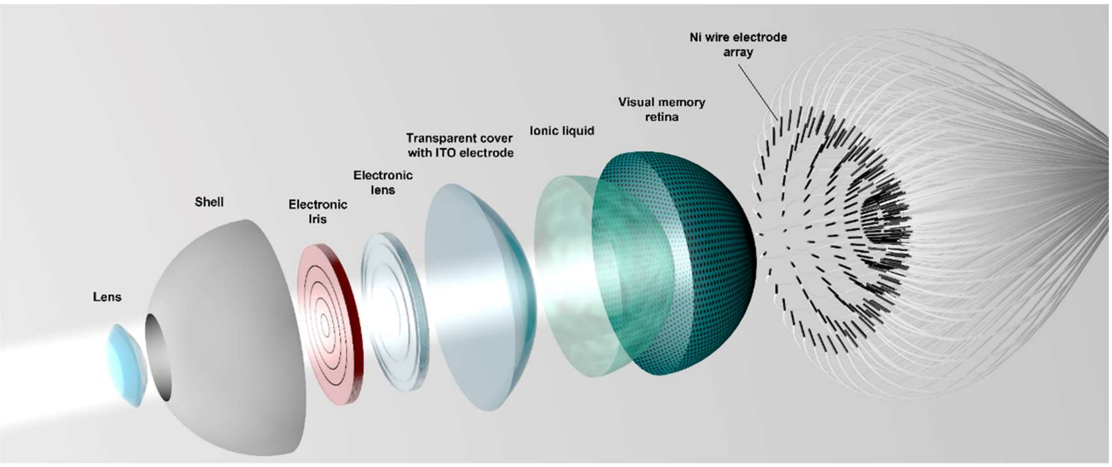


**Supplementary Fig. 11. Device structure of bionic eye**

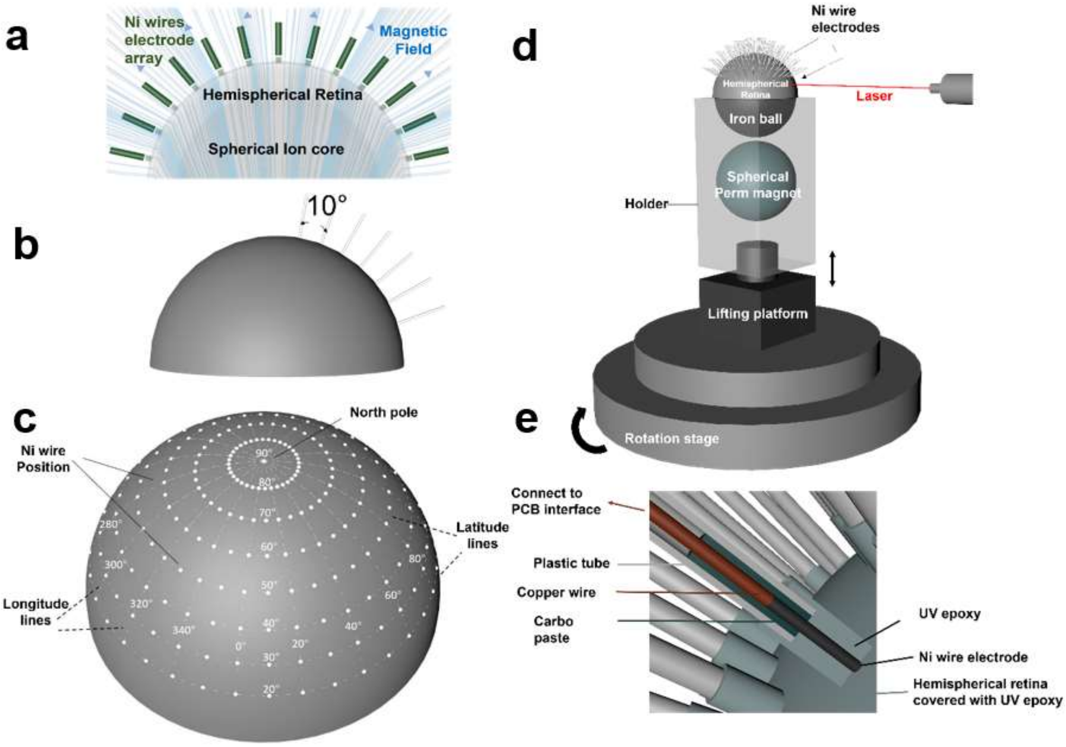


**Supplementary Fig. 12. Ni microwire electrodes assembling and pixel distribution**

**a**, Simulation of magnetic field during Ni wire electrodes alignment process. **b,** side view and **c,** Geographic coordinate system of Ni wires on hemispherical retina. **d,** Schematic of Ni wire electrodes alignment process. And **e,** detailed structure of Ni wire connected to PCB through a copper wire isolated by a plastic tube.

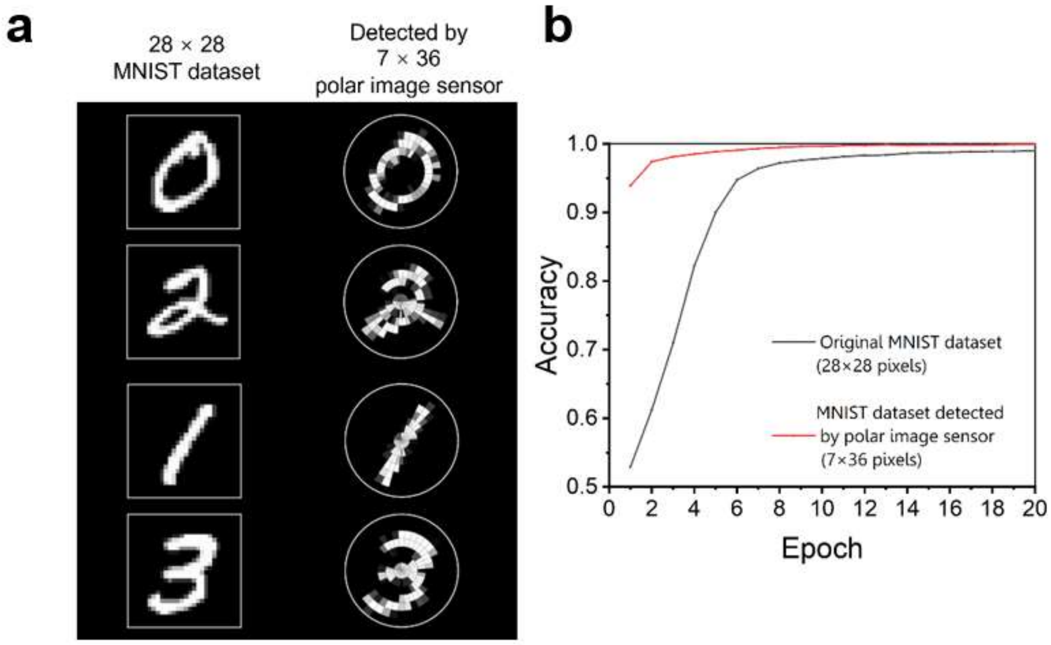


**Supplementary Fig. 13. MINST dataset and it detected by polar image sensor**

**a,** Image samples in MNIST dataset and it detected by hemispherical image sensor with polar pixel distribution. **b,** Two types of dataset recognized by an artificial neuron network. Note that polar distributed image sensor can get comparable recognition accuracy with less pixels.

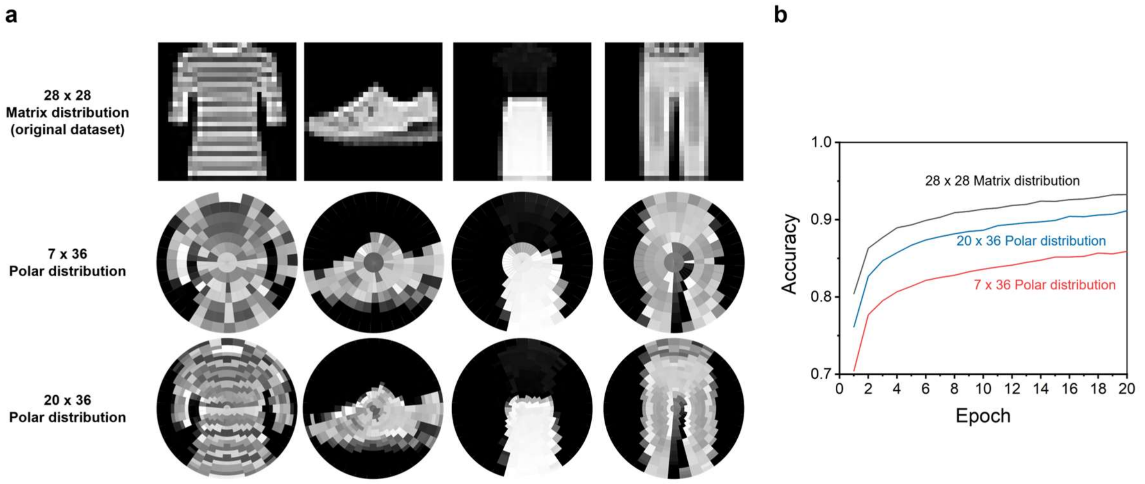


**Supplementary Fig. 14. Non-centered-oriented images reconstruction and related pattern recognition of image sensors with matrix and polar distribution**

**a,** Images reconstruction based on matrix and polar pixel distributions. And **b,** related identical curves of recognition accuracy after several epochs.

We not only simulated polar distribution with 7×36 pixels like our device, but 20×36 pixels (comparable to 28×28) as well.

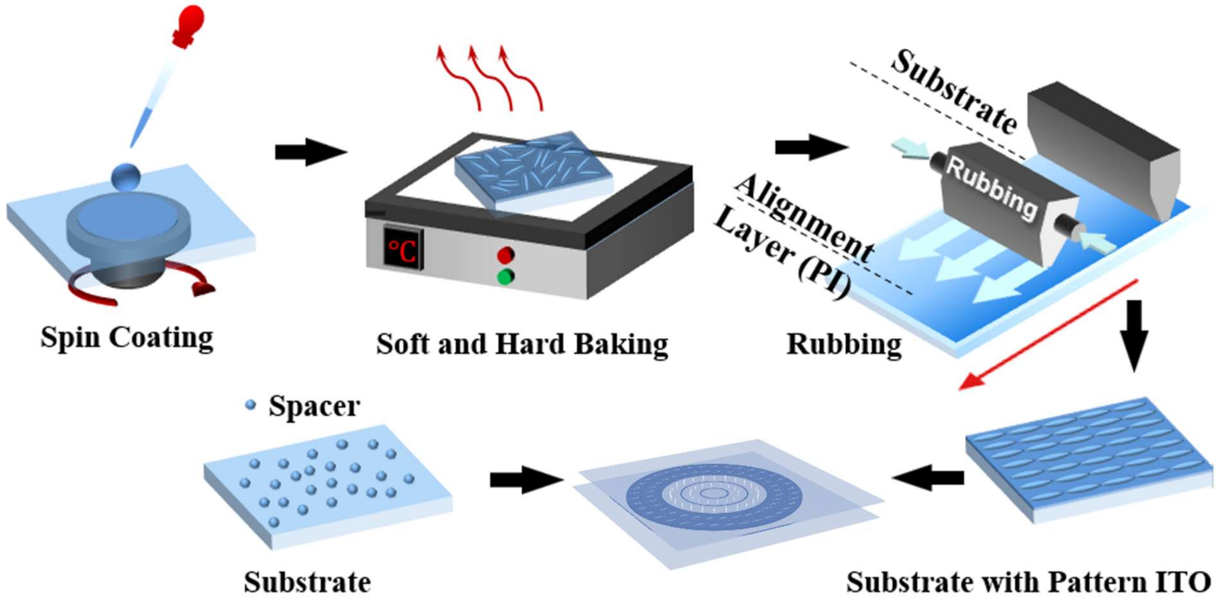


**Supplementary Fig. 15. Electronic Iris and the fabrication process**

The electronic iris is fabricated to achieve variable apertures from 3.14 mm$^2$ to 78.5 mm$^2$ in 5 steps. Moreover, the efficiency variance is analog. A patterned ITO with 5-concentric rings is fabricated using photolithography. The gap between the ring electrodes is 50 μm, which is small enough to meet the conditions in the near field of the eyes. Later, the patterned ITO glass and a common ITO glass are coated with a vertical PI and rubbed uni-directionally to provide the vertical alignment to the LC molecules. The LC was filled in the cell using the capillary action, which shows a pre-tilt angle of 88°. When the electric field is applied on different rings of the patterned ITO glass, the LC will show spatial rotation and analog efficiency tuning. The LC molecule is a negative liquid crystal that tends to align perpendicular to the direction of the electric field. The iris attains the maximum transmittance when the retardation of the liquid crystal matches the half-wave condition. The transmittance of the iris is zero when the voltage (V) is smaller than the threshold voltage ($V_{th}$) of the liquid crystals. With a gradually increasing electric field ($V > V_{th}$), the liquid crystal molecules lie down, and so the iris shows transmittance. Like a biological iris, this artificial iris can control the amount of light reaching the retina by selecting the number of rings in the transparent state.

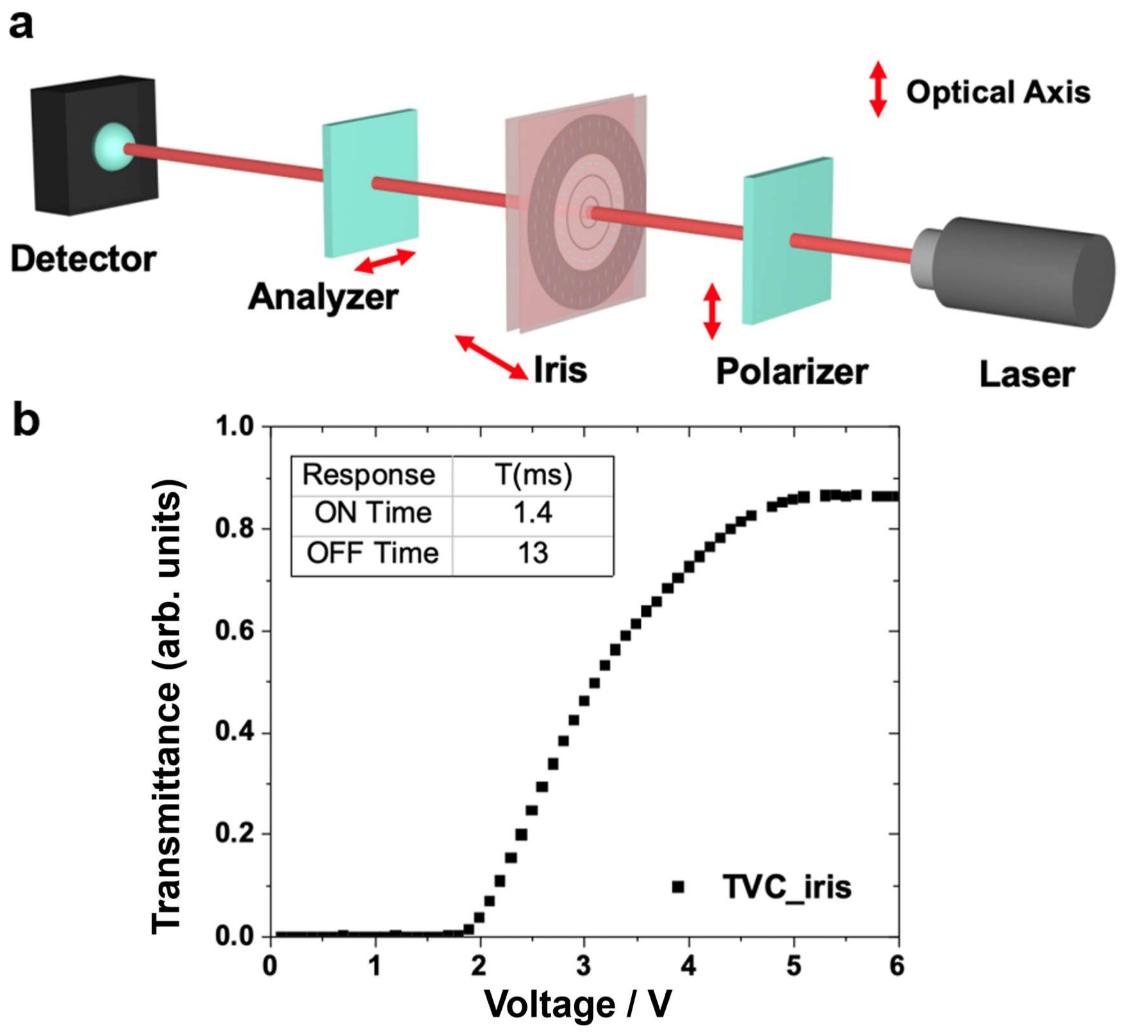


**Supplementary Fig. 16. Electronic Iris performance characterization**

**a**, Schematics of Measurement setup for EO performance of LC iris and **b**, the transmittance versus voltage curve of LC iris.

The EO performance of the LC iris is recorded between two crossed polarizers for red incident light. Fig. S10 shows the EO response of the single liquid crystal iris. The total response is $T_{on}$+$T_{off}$ = 14.4ms. We used high birefringence negative liquid crystal LEG646 (Slichem LC Material Co.Ltd) to achieve a high contrast ratio and wide viewing angle. The designed Iris shows continuous efficiency tuning from 0 to 87% within 5V.

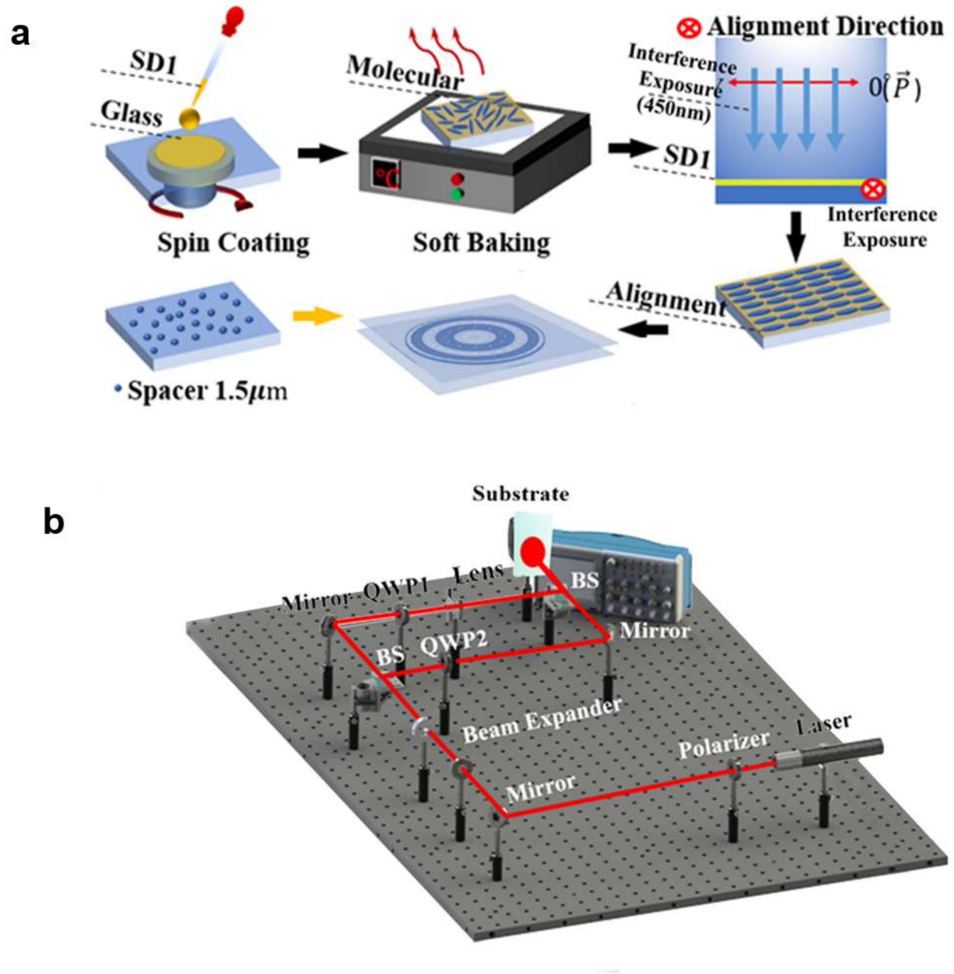


**Supplementary Fig. 17. Electronic lens and the fabrication process**

Schematics of **a,** fabrication process, and **b,** The exposure setup based on Mach-Zehner Michelson interferometer for alignment of Pancharatnam−Berry (PB) lens

The liquid crystal Pancharatnam-Berry (PB) lens is an optical phase modulation device wherein the liquid crystal's director orientation continuously changes in the azimuthal plane to modulate the light beam. The PB lens can change the lens polarity by merely adjusting the polarization of the incident light. The PB lens has a small form factor and high flatness, which offers the maximum aperture ratio and the largest aperture.

Alike the human crystalline lens, which can change the focal length by changing the lens thickness, the focal length of the electronic lens can be switched by an electric signal. We used photo-alignment to fabricate the switchable liquid crystal PB lens. The photo-aligned sulfuric azo-dye (azo-dye SD1) is used to align the LC director in the PB lens. First, the SD1 is coated onto the ITO-coated glass plate and then irradiated with the interference pattern required for the PB lens. We used interference of two orthogonal circularly polarized light beams to achieve the PB lens profile. After passing through the PB lens, the light sees spatial phase difference

same as the lens. Later, we sandwiched the LC in between the two photo-aligned glass plates. For the applied voltages ($V$) smaller than the threshold voltage ($V_{\mathrm{th}}$) of the liquid crystal, the phase profile of the lens is maintained. However, for $V > V_{th}$ (in the range of 3-5 V), the positive LC molecule follows the direction of the electric field, and the phase profile is distorted and eventually disappears.

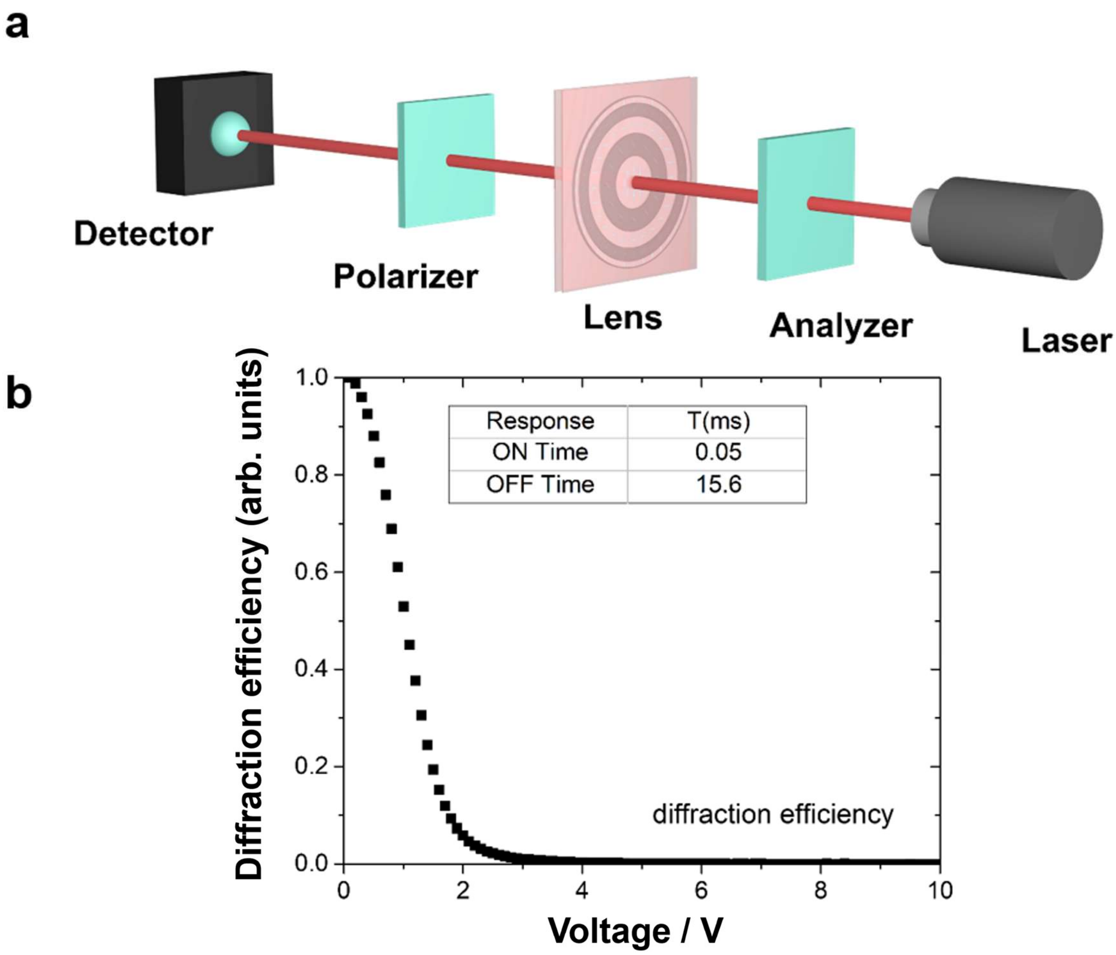


**Supplementary Fig. 18. Electronic lens performance characterization**

**a,** Schematics of Measurement setup for EO performance of LC lens and **b,** the diffraction efficiency versus voltage curve of LC lens

The EO performance of the PB lens is recorded between two crossed polarizers for the red ($\lambda = 632nm$) incident light. The total response is $T_{on} + \mathrm{T}_{off}$ = 15.65 ms. We used positive liquid crystal E7 (Merck) for the PB lens. Small focal length (50 mm) and large numerical aperture (0.5) are achieved to fulfill the requirement of the super eye when combined with a fixed glass lens(f=23mm). All object distances from 30 mm (2f) to infinity show an effective imaging process on the detector array.

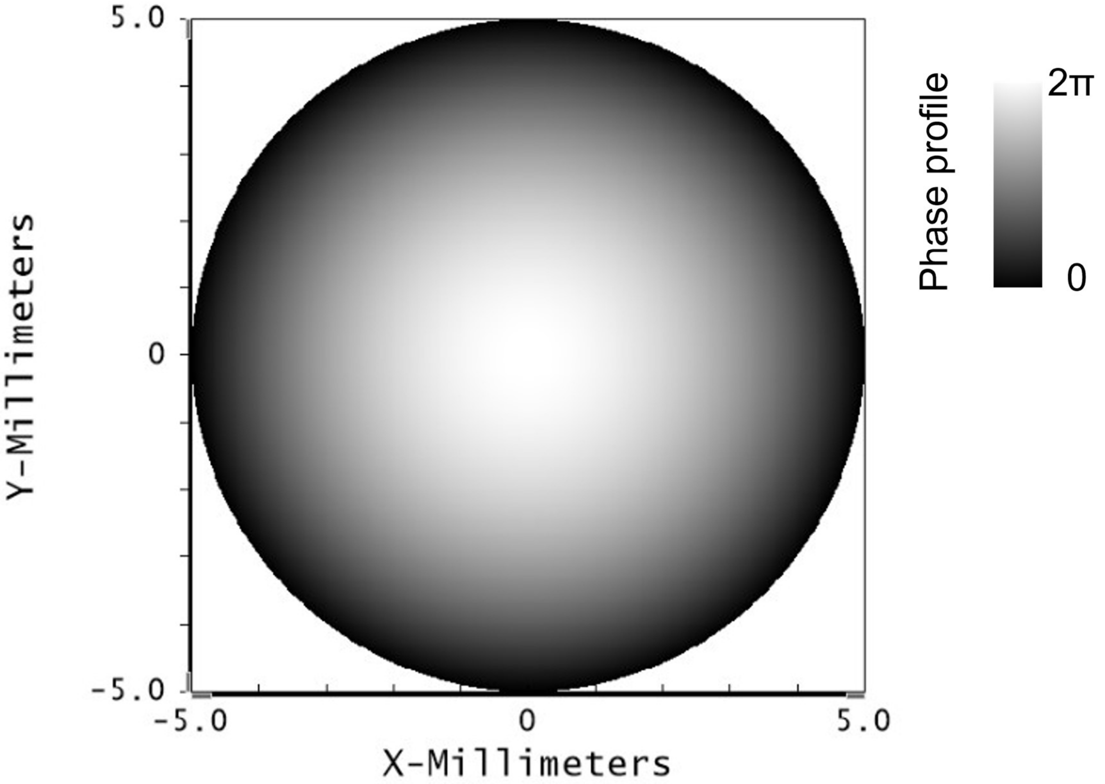


**Supplementary Fig. 19. Phase distribution of the artificial crystalline lens.**
The diameter of the lens is 1 cm.

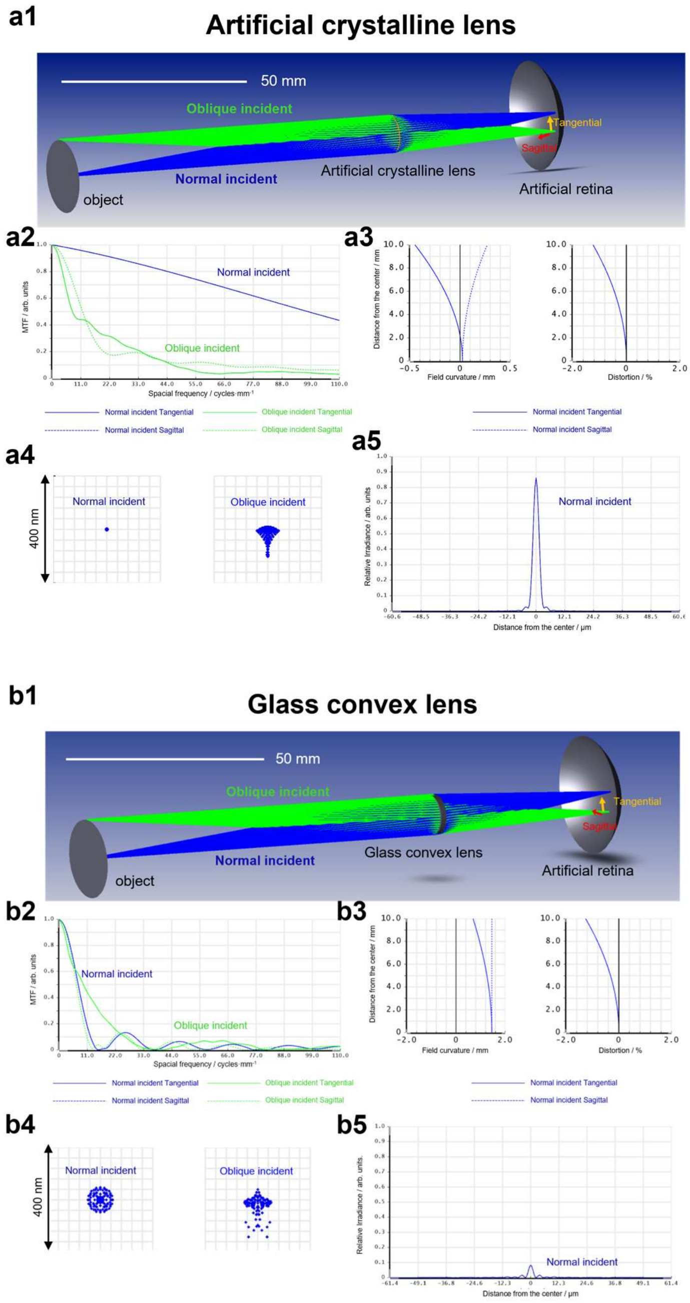


**Supplementary Fig. 20. Optical properties of artificial crystalline lens and conventional glass lens**

Optical simulation of **a1-a5,** artificial crystalline lens and **b1-b5**, a conventional glass lens. **a1, b1,** the schematic of optical path in the simulation. **a2, b2,** MTF – special frequency curves. **a3, b3,** Field curvature and distortion curves. **a4, b4,** focal points distribution patterns. **a5, b5,** spatial distribution of irradiance close to focal point.

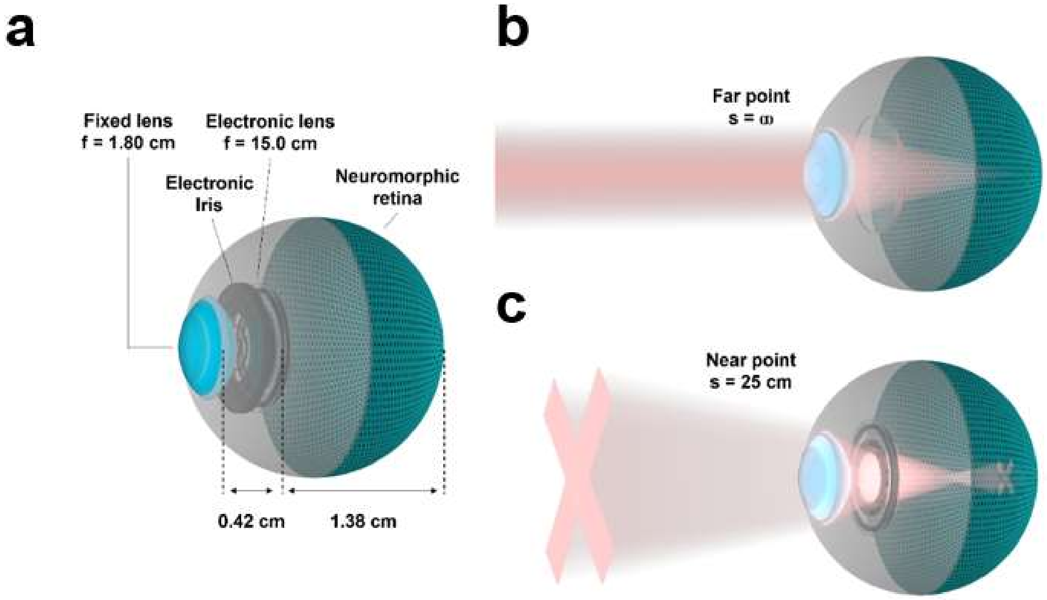


**Supplementary Fig. 21. Near point and far point conditions of the spherical eye**
**a,** Schematics of the optical sub-system and the artificial retina. **b,** Far point condition and **c,** Near point condition of the optical subsystem.

Far point condition:

While the focal length of the electronic lens switch to ∞, the focal length of the system

$f = f_{fixed\ lens} = 1.80$ cm

Image distance $v = 1.80$ cm

According to

$$\frac{1}{u}+\frac{1}{v}=\frac{1}{f}$$

Where u is object distance

Far point $s = u = \infty$

Near point condition:

While the focal length of the electronic lens switch to 5 cm, the focal length of the system

$f = \frac{f_{fixed\ lens} \times f_{electronic\ lens}}{f_{fixed\ lens} + f_{electronic\ lens} - D} = 1.6483$ cm

Where D is the distance between fixed lens and electronic lens, $D = 0.42$ cm

Image distance is the distance between back principal plane and the north pole of the retina.

$v = d + D * \frac{f}{f_{fixed\ lens}} = 1.7646$ cm

where d is the distance between electronic lens and the center of the retina, $d = 1.38$ cm

Near point distance $s = u = 25.00$ cm

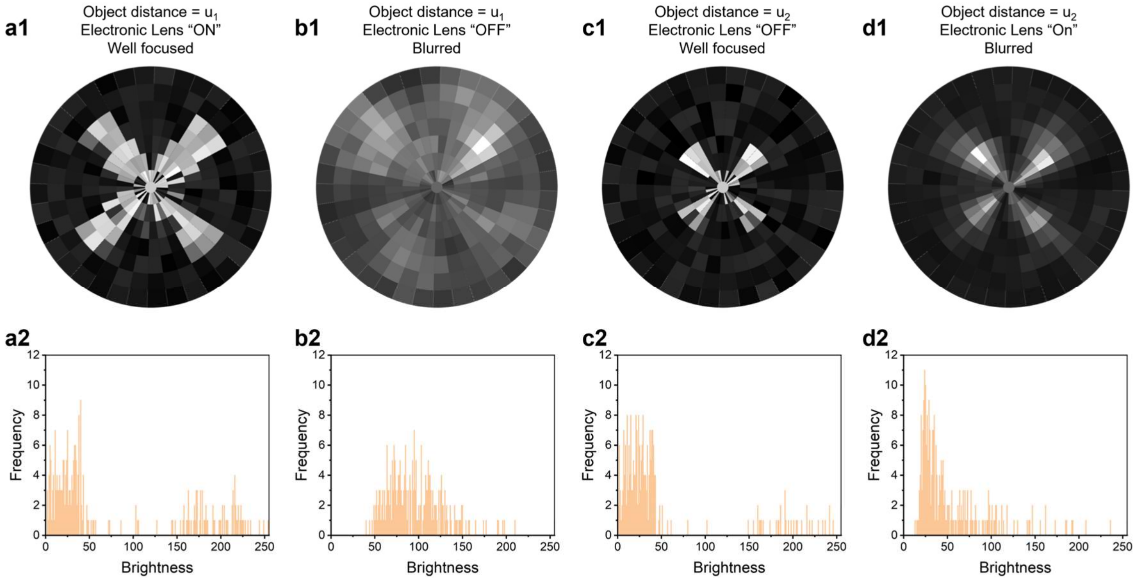


**Supplementary Fig. 22 The comparation of well-focused and blurry images.**

Well focused and blurry images of **a1-b1**, closed object (Object distance = $u_1$) and **c1-d1**, far away object (Object distance = $u_2$). And **a2-d2**, related brightness distribution.

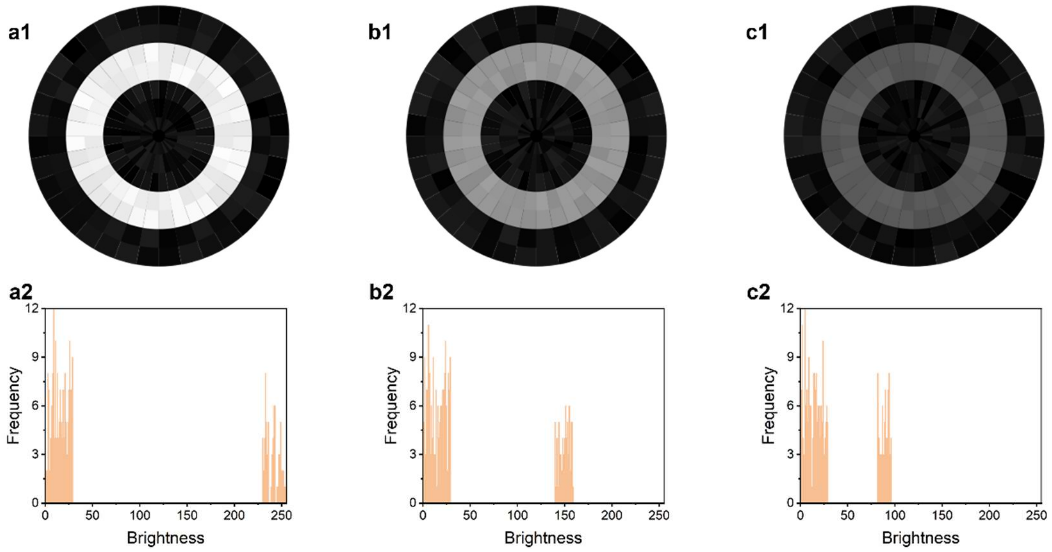


**Supplementary Fig. 23 Pattern reconstruction with different electronic iris states.**
**a1-c1**, "O" shape reconstruction with different electronic iris state and **a2-c2**, related brightness distributions.

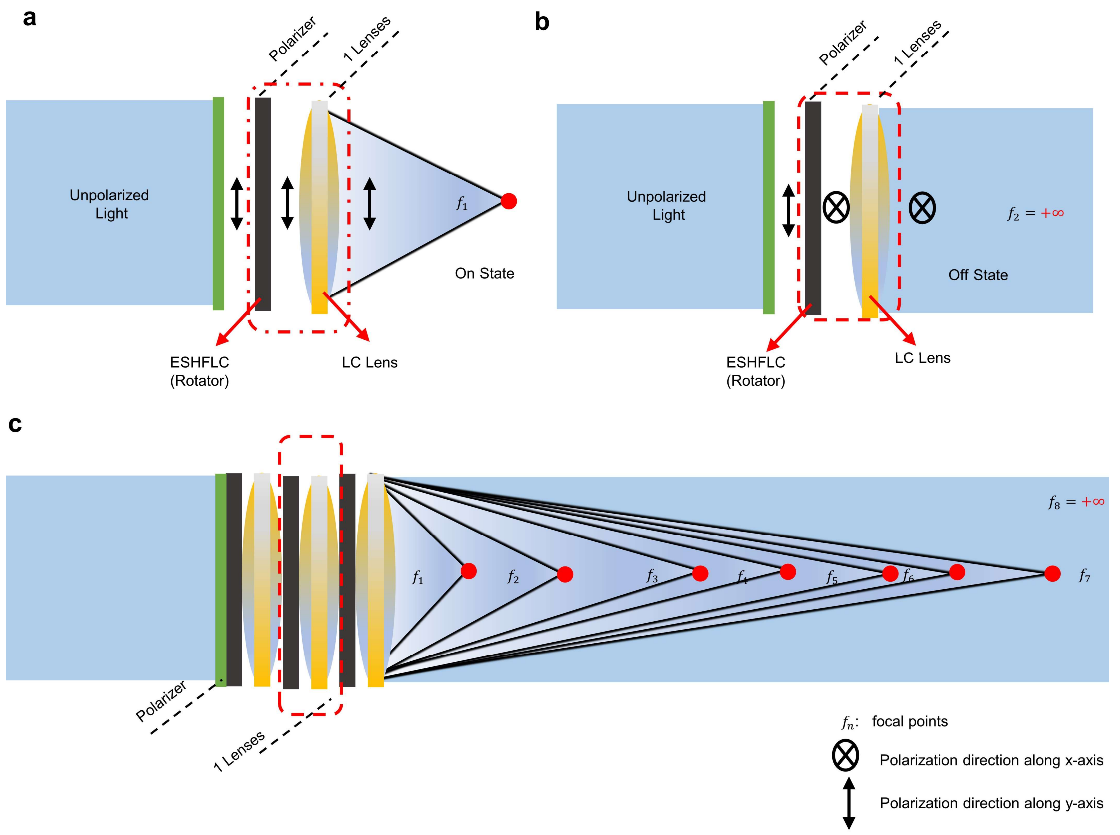


| State of each lens | | | System focal length |
|---|---|---|---|
| **$f_{c1}$** | **$f_{c2}$** | **$f_{c3}$** | |
| on | off | off | **$f_1$** |
| on | off | on | **$f_2$** |
| on | on | off | **$f_3$** |
| on | on | on | **$f_4$** |
| off | off | on | **$f_5$** |
| off | on | off | **$f_6$** |
| off | on | on | **$f_7$** |
| off | off | off | **$f_8$** |

**Supplementary Fig. 24 Three electronic lenses based imaging system with close to continuous focal length switching.**

The schematics of **a,** An electronic lens at "ON" state. **b,** An electronic lens at "OFF" state. And **c,** a 3 electronic lenses based optical system with 8 focal points.

**Supplementary Table 1 Comparation of commercial and our tunable lens systems**

| Tunable lens systems | Number of lenses | Diameter and length / mm | Weight / g | Focal length range / mm | FoV | Switch type | Switch time |
|---|---|---|---|---|---|---|---|
| **Our system** | **2** | **Φ10 × 10** | **<20** | **25 & ∞** | **>140°** | **Electrical** | **~5 ms** |
| Canon RF100-500mm F4.5-7.1 L IS USM | 20 | Φ93.8 × 207.6 | 1370 | 100-500 | 24° | Mechanical | >100 ms |
| NIKKOR Z 100-400MM F/4.5-5.6 VR S | 25 | Φ98 × 222 | 1355 | 100-400 | 24° | Mechanical | >100 ms |
| FUJINON GF20-35mmF4 R WR | 14 | Φ88.5 × 112.5 | 725 | 20-35 | 108° | Mechanical | >100 ms |
| Sony FE PZ 16-35mm F4 G | 13 | Φ80.5 × 88.1 | 353 | 16-35 | 107° | Mechanical | >100 ms |
